# SAR-MLD1-2025-MangoLeaf: A comprehensive high-quality mango leaf dataset for disease classification

**DOI:** 10.1016/j.dib.2026.112826

**Published:** 2026-05-07

**Authors:** Md. Robiul Awoal, Afjal H. Sarower, Md. Zubair Islam, Sharmin Akhter, Md. Jubayer Hossain, S.M. Aminul Haque, Sheak Rashed Haider Noori

**Affiliations:** a Department of Computer Science and Engineering, Daffodil International University, Dhaka, Bangladesh; b SAR Lab, Department of Computer Science and Engineering, Daffodil International University, Dhaka, Bangladesh

**Keywords:** Mango leaf, Disease classification, Machine learning, Computer vision

## Abstract

This article presents a carefully curated dataset of mango leaves, collected from the northern regions of Bangladesh, specifically from Naogaon District, a major mango-producing area. Bangladesh, known for its agriculture, frequently faces leaf diseases that impact mango yield and quality. Early detection is crucial to prevent widespread damage and ensure better disease management. The dataset comprises 4921 raw image samples, categorized into five distinct classes: Healthy, Anthracnose, Powdery Mildew, Turning Brown, and Gall Midge. The images were captured under natural lighting conditions to preserve the leaves' intrinsic visual features, ensuring authenticity and variability. This dataset is a valuable resource for botanical research and machine learning applications, particularly in the automated classification of mango leaf diseases, helping researchers develop more effective disease detection models.

Specifications Table

**Table:** 

Subject	Precision Agriculture
Specific subject area	Artificial Intelligence, Computer Vision, Leaf disease classification using deep learning.
Type of data	Digital Images
Data collection	The dataset was collected from the Naogaon district in northern Bangladesh, a region recognized as one of the world’s prominent mango-producing areas. Mango orchards were selected to represent different mango varieties. After identifying infected trees, all leaves exhibiting visible disease symptoms were first collected from the trees, and images were subsequently captured against a white background. In total, 5100 images were acquired, of which 4921 passed quality control and were deemed suitable for deep learning applications in leaf disease classification.
Data source location	Two of the following mango orchards were selected for data collection: Vai Vai Fruit Farm, Mohadevpur, Naogaon, Rajshahi (24.950595, 88.748189) Ma Mango Garden, Sapahar, Naogaon, Rajshahi (25.120411, 88.564797)
Data accessibility	Repository name: Mendely Data identification number: part 1: 10.17632/sd8hzpg69b.4 part 2: 10.17632/j3bn63t4sp.4 Direct URL to data: part 1:https://data.mendeley.com/datasets/sd8hzpg69b/4 part 2: https://data.mendeley.com/datasets/j3bn63t4sp/4
Related research article	N/A

## 1 Value of the data

This dataset holds significant value for advancing agricultural research and precision farming, particularly within mango cultivation. It is a crucial resource for research, development, and practical applications. With the dataset we make the following contribution-

• The dataset comprises five distinct classes representing mango leaf conditions, including two disease categories (anthracnose and powdery mildew), as well as healthy leaves, abiotic stress (leaf browning), and insect-induced galls (Golmichi/Gall midge). All images are meticulously labeled and verified under the supervision of an agricultural expert.
• A total of 4921 raw images were meticulously labeled and ground-truthed into five distinct classes under the direct supervision of an agricultural expert.
• This high-quality dataset serves as a valuable resource for computer vision and precision agriculture applications, enabling the development and evaluation of robust mango leaf disease classification models. Its diversity of classes and intra-class variability further support broader applicability and reproducible research for reliable, high-impact solutions.

## 2 Background

Mango (Mangifera indica) ranks as the ninth most widely cultivated fruit in the world, with its peak harvest season occurring from March to August in the South Asia region. As such, it plays a vital role in Bangladesh’s agriculture [1]. The northern region, particularly Naogaon, is renowned for its experienced cultivators, who have been cultivating high-quality local mango varieties such as Amrupali, Fazli, Langra, and Himsagar for decades. With an annual mango production exceeding 2.5 million metric tons, the cultivation of mangoes supports the livelihoods of millions of farmers, contributing significantly to the nation's economy. However, mango trees are susceptible to various leaf diseases, which negatively impact both fruit quality and overall productivity [2].

Historically, disease detection in mango crops has depended on visual examination by experts, a process that is both time-consuming and prone to human error. Recent advancements in deep learning (DL) and machine learning (ML) have presented automated disease detection as a promising alternative, offering quicker and more accurate diagnoses [3]. These advanced techniques require large, high-quality datasets to train models capable of identifying subtle changes in leaf appearance caused by diseases. While several datasets for mango leaf disease classification exist, many have limitations, such as a small number of images per disease class or insufficient representation of the diversity of diseases that affect mango plants. Our dataset addresses these issues by providing a larger and more representative collection of images, thereby enabling the development of more reliable and precise machine learning models. We anticipate that this dataset will be a valuable resource for ongoing efforts to automate disease diagnosis in mango crops, ultimately leading to increased yields and improved protection of the livelihoods of Bangladeshi farmers [4].

## 3 Data Description

The dataset includes a series of images with varied severity of mango leaf diseases. A total of 5100 images were collected from harvested leaves and classified into five distinct categories. All images maintain a uniform resolution of 640×480 pixels. Data acquisition occurred between **December 2023 and April 2024** through meticulous field surveys, guided by local farmers and domain experts. Following quality control measures, including the removal of poor-quality and redundant images, the final, curated dataset size is 4921 images. Table 1; 2.

**Table 1:** Number of images per class in the mango leaf disease dataset.

Sample Label	Folder Name	Original Image	Causal Agent (Scientific Name)
1	Anthracnose	972	Colletotrichum gloeosporioides
2	Powdery Mildew	983	Oidium mangiferae
3	Turning Brown	1000	Physiological / abiotic stress
4	Gall Midge	983	Procontarinia mangiferae
5	Healthy	983	-
Total		4921

**Table 2:** Sample mango leaf images across the five disease classes.

Serial No.	Disease Name	Sample images	Disease Description
1	Anthracnose		**Anthracnose** [5] is a fungal disease caused by Colletotrichum gloeosporioides. It typically begins as small water-soaked spots on mango leaves during humid or rainy conditions, spreading rapidly through moisture. Over time, these spots enlarge into irregular dark brown to black patches that may merge, often surrounded by yellow halos. Anthracnose is highly destructive, especially in humid climates, affecting leaves, flowers, and fruits, and can cause premature leaf drop and significant crop loss.

2	Gall Midge (Insect Galls)		Mango Leaf **Gall Midge** [6], caused by the insect Procontarinia mangiferae, leads to the formation of small, wart-like galls or swellings on the upper surface of mango leaves. The larvae of the gall midge induce abnormal growth in leaf tissues, resulting in distorted, thickened, and curled leaves. These galls are often blackish or dark and contain the developing larvae inside. Infestations can reduce photosynthetic area, and weaken the overall health and productivity of the mango tree if not managed properly.

3	Turning brown		Turning brown [7] disease in mango leaves refers to browning caused by environmental stress factors such as sunburn, nutrient deficiencies, water stress, or aging. It often starts with discoloration at the leaf tips or margins, which gradually expands to larger brown dry patches. Unlike specific pathogenic diseases, this condition reflects general plant stress and usually results in mild to moderate damage that can reduce overall plant vigor.

4	Powdery mildew		Powdery mildew [8], caused by the fungus Oidium mangiferae, usually infects young leaves and tender shoots under cool, dry mornings followed by warm days. The infection appears as a white, powdery fungal growth that covers the leaf surface. Infected leaves may curl, dry out, and drop prematurely, leading to moderate to severe damage, particularly reducing flowering and fruit set if left untreated.

5	Healthy		A healthy mango leaf exhibits a uniform green color with no spots, deformations, or discolorations. It has a smooth surface with clearly visible veins and normal thickness, indicating balanced nutrients, adequate moisture, and freedom from pest or pathogen attacks. Healthy leaves represent the normal baseline condition in mango leaf health assessment.

**Fig. 1:**
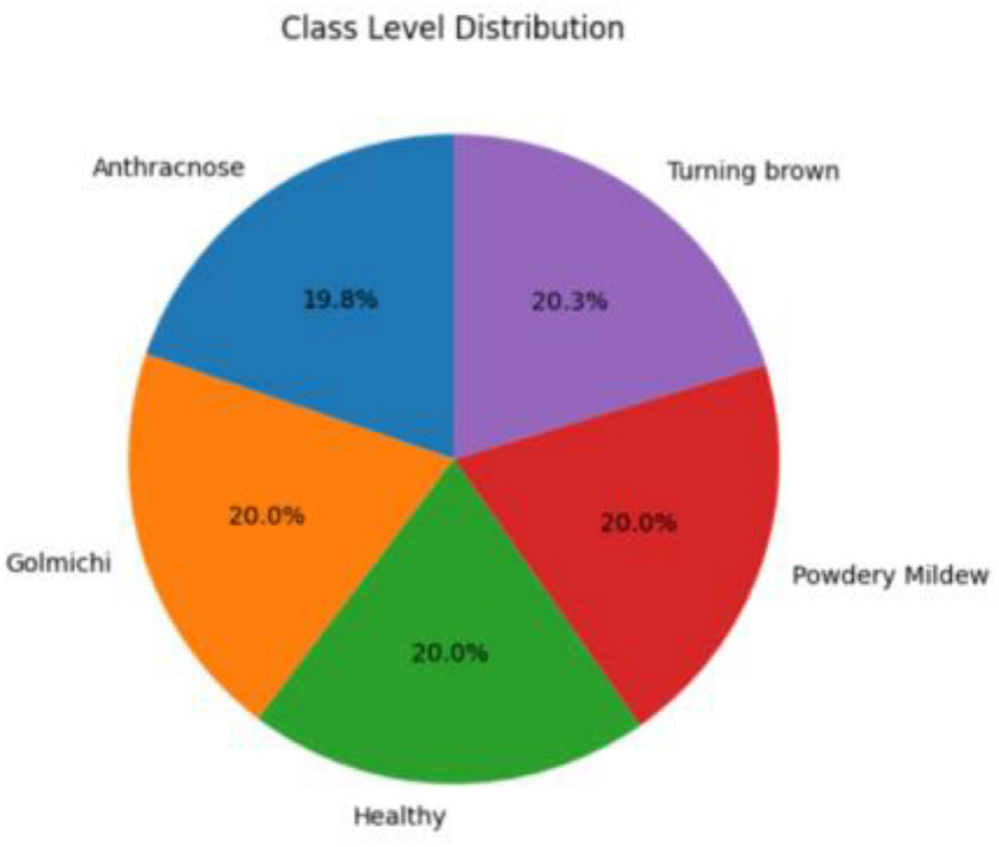
Dataset class distribution.

To capture the full natural variation of the disease, images were collected under a wide range of environmental conditions, including varying light, humidity, and temperature. For standardization, photographs of both the top and bottom of each infected leaf were taken against a white background. This deliberate choice of a white backdrop was critical for isolating the leaf features, enabling precise disease signature extraction and reducing noise for machine learning applications. Despite challenges like fluctuating light intensity and potential background noise, the primary goal was met: to capture high-quality images that accurately represent the distinctive signs of each mango leaf disease class. Fig. 1 illustrates the distribution of images across the five distinct classes, demonstrating a nearly equal ratio that results in a balanced dataset.

## 4 Experimental Design, Materials and Methods

**Fig. 2:**
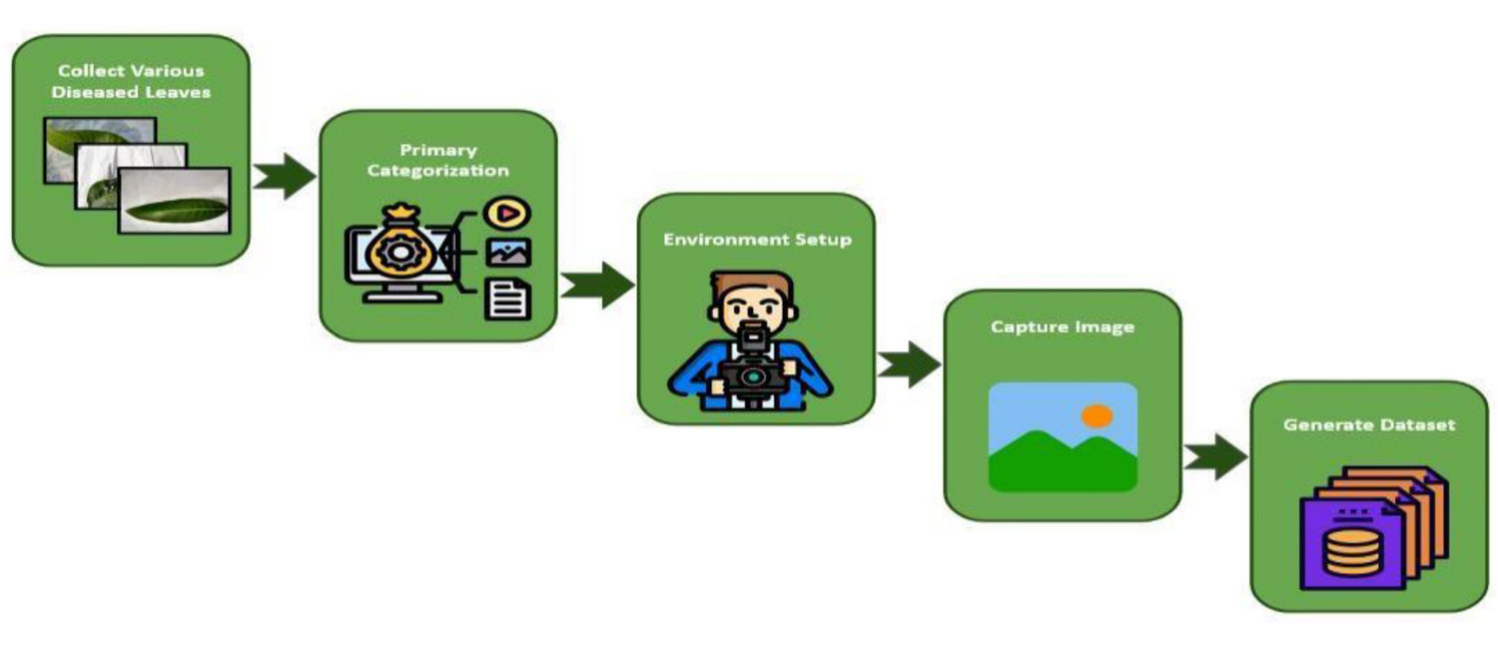
Workflow for the mango leaf disease dataset generation.

The process of acquiring the mango leaf disease dataset was a well-organized workflow illustrated in Fig. 2. The process began with the careful selection of individual mango leaves from each diseased tree to ensure an equal representation of various stages of disease. To ensure dataset diversity, leaves were randomly selected from multiple branches and parts of each tree.

The leaves of 5 different classes; Anthracnose, Powdery Mildew, Turning brown, Insect Galls and Healthy were and domain expert ensured every kind was actually of that nature. A plant disease specialist from the Upazila Agricultural Office in Mohadevpur, Naogaon, who is involved in both research and teaching, examined the leaves initially. Using accepted field pathology sources, the diagnosis was made based on the symptoms that were readily apparent. Following harvesting, leaves were stored in separate, temperature-controlled vessels to prevent any deterioration before imaging. To confirm that we imaged the features of the leaf diseases correctly, we examined each leaf for disease discoloration, lesions, and other manifestations of the disease very carefully.

We positioned the camera equipment at approximately 6 inches above the leaf platform and used a 5x zoom lens to record high-resolution, clear images. A solid white background was used consistently for better contrast, and a series of photographs from various angles were captured in an attempt to cover a complete picture of the health of the leaf in a detailed manner. After capturing each leaf, the images were transferred from the internal storage of the smartphone to an external hard disk so that they could be processed later. The images were checked for correct classification by the domain expert prior to being placed in respective folders according to the different categories of plants. Once processing of each batch of images was completed, data from the last batch of images was erased from the memory in the smartphone to create space for capturing another batch. This continued in an orderly manner until photographs for all types of plants were captured and arranged in chronological order.

### 4.1 Camera specification

For our data collection, we captured images of mango leaf diseases using two different smartphone cameras: the Realme 5i and the Samsung Galaxy J5 Prime (SM-G570F). The Realme 5i, equipped with a 12 MP primary rear camera and an 8 MP ultra-wide lens, provided clear and detailed images with a versatile quad-camera setup, making it suitable for capturing both close-up and wide-angle shots. The Samsung Galaxy J5 Prime, also offered decent image quality with its 13 MP rear camera. Both devices were used to document various symptoms of mango leaf diseases, enabling a comparative analysis of their image-capturing capabilities for agricultural diagnosis. The ISO is set between 135 and 145 mm to compensate for lighting, and the exposure value (EV) is +2 EV to lighten images when necessary. The shutter speed is 1/49 s to give well-exposed images in normal lighting. The camera digitally zooms 5x for close-up shots, and it is positioned around 6 inches from the object for capturing high-resolution photos of the mango leaves. The settings ensure that the images have consistent quality used in training and testing the model for disease diagnosis. All the information is given in Table 3.

**Table 3:** Camera setup details.

Camera Type	Wide Camera, 28 mm
Focal Length (f)	1.8
Brightness	Studio Light
Resolution	8 MP
ISO	135–145
Shutter Speed	1/49s
Exposure	2 EV
Zoom	5X
Camera Distance from Object	6 inches (≈ 0.152 m)

## Limitations

The mango leaf disease dataset has several limitations. It covers only a subset of mango leaf diseases and is region-specific, which may limit its ability to generalize to other conditions. The images were captured under natural lighting, leading to potential inconsistencies in illumination. Additionally, the dataset focuses on specific disease stages and ignores seasonal variations, while only considering visual features and not incorporating other data, like chemical composition or environmental factors, that could improve disease identification accuracy.

## Ethics Statement

This paper does not involve any research involving human or animal subjects by any of the authors. Datasets available publicly were utilized in this paper. Application of the datasets used here must adhere to good citation practices.

## CRediT Author Statement

Md. Robiul Awoal: Conceptualization, Methodology, Data Collection, Investigation; Afjal H. Sarower: Supervision, Methodology, Writing – review & editing; Md. Zubair Islam: Conceptualization, Software, Data Collection; Sharmin Akhter: Software, Data curation, Writing – original draft; Md. Jubayer Hossain: Softwar, Resources, Data curation; S.M. Aminul Islam: Project administration, Writing – review & editing. Sheak Rashed Haider Noori: Validation, Writing – review & editing.

## Data Availability

Mendeley DataSAR-MLD1-2025: A High Quality Mango Leaf Dataset for Disease Classification (Original data)
Mendeley DataSAR-MLD1-2025: A High Quality Mango Leaf Dataset for Disease Classification (Original data)
